# (Dis)connected by design? Using participatory citizen science to uncover environmental determinants of social connectedness for youth in under-resourced neighbourhoods

**DOI:** 10.1186/s12889-024-20597-4

**Published:** 2024-11-11

**Authors:** Meridith Sones, Meg Holden, Yan Kestens, Abby C. King, Mimi Rennie, Meghan Winters

**Affiliations:** 1 Faculty of Health Sciences, Simon Fraser University, 8888 University Drive, Burnaby, BC V5A 1S6 Canada; 2 Urban Studies and Resources and Environmental Management, Simon Fraser University, 515 W Hastings St, Vancouver, BC V6B 5K3 Canada; 3 École de santé publique, Université de Montréal, 7101 Park Ave, Montreal, QC H3N 1X9 Canada; 4 Department of Epidemiology & Population Health and the Stanford Prevention Research Center, Department of Medicine, Stanford University School of Medicine, 1701 Page Mill Road, Palo Alto, CA 94304-1210 USA; 5 South Vancouver Neighbourhood House, 6470 Victoria Dr, Vancouver, BC V5P 3X7 Canada

**Keywords:** Youth, Social connectedness, Built environment, Social infrastructure, Citizen science, Participatory action research

## Abstract

**Background:**

Social isolation and loneliness are a growing public health concern. Inadequacies in neighbourhood social infrastructure can undermine social connectedness, particularly for youth, who are dependent on their local environments yet often marginalized from public spaces and city planning. Integrating citizen science with participatory action research, the Youth.hood study set out to explore how neighbourhood built environments help or hinder social connectedness from the understudied perspective of youth in under-resourced and racialized communities.

**Methods:**

Youth (*n* = 42) from three neighbourhoods in Vancouver, Canada were recruited to: (1) Assess environmental assets and barriers to connectedness in their neighbourhoods using a digital photovoice app; (2) Analyze and prioritize their collective data into themes; and (3) Design and advocate for environmental improvements through a participatory workshop and forum with residents, city planners, and elected officials. Data on participant characteristics and neighbourhood perceptions were collected via an online survey and analyzed descriptively. Participatory analysis was conducted with youth using methods from thematic analysis, photovoice, and design thinking.

**Results:**

Youth captured 227 environmental features impacting their connectedness. The most frequently reported assets were parks and nature (*n* = 39, 17%), including formal and informal green spaces, and food outlets (*n* = 25, 11%). Top barriers included poor neighbourhood aesthetics (*n* = 14, 6%) and inadequate streets and sidewalks (*n* = 14, 6%). Thematic analysis with youth underscored four themes: (1) Connecting through mobility: The fun and functionality of getting around without a car; (2) The power of aesthetics: Mediating connections to people and place; (3) Retreating to connect: Seeking out social and restorative spaces for all; and (4) Under-resourced, not under-valued: Uncovering assets for sociocultural connection. Youth described their local environments as affording (or denying) opportunities for physical, emotional, and cultural connection at both an individual and community level.

**Conclusion:**

Our findings extend evidence on key environmental determinants of social connectedness for youth, while highlighting the potential of community design to support multiple dimensions of healthy social development. Additionally, this work demonstrates the resilience and agency of youth in under-resourced settings, and underscores the importance of honouring assets, co-production, and intergenerational planning when working to advance healthy, connected, and youthful cities.

**Supplementary Information:**

The online version contains supplementary material available at 10.1186/s12889-024-20597-4.

## Introduction

Social connectedness is a key element of healthy and sustainable cities. A multifaceted concept, it encompasses the interactions, relationships, roles, and sense of connection experienced by individuals or communities [1–3]. Evidence on the health impacts of social connectedness is considerable and reports multiple physical, mental, and cognitive effects across the life span [1]. During adolescence, social connectedness is associated with improved wellbeing, while social isolation and loneliness are risk factors for depression and anxiety [4, 5]. Additionally, youth in communities where neighbours interact, support, and trust each other report better self-reported physical and mental health, and feelings of safety and self-esteem [6–8]. 

Trends suggest that social connectedness among young people has been declining and exacerbated by the COVID-19 pandemic. Youth and young adults report weaker sense of belonging and trust than other age groups and experience some of the highest rates of loneliness globally [9]. In 2021, nearly one in four youth and young adults in Canada reported feeling lonely always or often, close to double the population rate [10]. Other factors can intersect with age to put certain people more at risk of social isolation, for example, racialized youth or those living in lower income households [2, 11]. 

Researchers, practitioners, and governments—along with young people themselves—underscore the need to address the epidemic of loneliness and social isolation with intersectoral action that prioritizes equitable and participatory city building [1, 2, 5]. Strengthening social infrastructure, including the built or physical elements of a community that support social connection, can grow healthier and more sustainable cities at a population scale [12–15]. However, lack of evidence on how to design youth-inclusive communities is a major limitation to leveraging the full potential of place-based interventions to improve social connection outcomes for adolescents [5, 16]. 

### Built environment and youth social connectedness

The influence of neighbourhoods on the social wellbeing of adolescents is understudied in built environment research. The majority of studies examine physical health outcomes and many concentrate on school or residential settings, leaving sparse evidence on how neighbourhoods facilitate youth social connectedness [16–18]. General population studies associate higher levels of social interaction, neighbourhood ties, and sense of belonging with features of “complete communities”, where destinations like high quality parks and public spaces, recreational facilities, shops, and services are close to home and easily reached by walking, cycling, or public transit [11, 19–23]. The limited youth-focused research available reinforces the importance of local destinations and services, freely accessible via safe and active travel [16, 17, 24–29]. 

Despite the potential contribution to the objectives of complete communities, urban planning rarely accounts for the social and developmental needs of youth in neighbourhood built environments. Youth naturally spend more time in their neighbourhoods and are more dependent on their local environments [25]. Yet, outside of token amenities like skateparks [30–32], youth are often excluded or alienated from neighbourhood spaces—both formally through restrictive policies and design, and informally through social policing tied to the public image of teenagers as “troublemakers” [31, 33, 34]. Perceived as too old for playgrounds, too young to afford commercial spaces, and given little opportunity to participate in decisions that shape their neighbourhood, youth are made to feel invisible in the formal public realm and left to “claim the leftovers” when seeking spaces for connection [25]. 

For some youth, access to the city is further undermined by other socio-structural barriers that intersect with age, including gender, race, and socioeconomic status [35]. Inequities in the distribution and quality of social infrastructure persist in urban areas. Access to neighbourhood green spaces in many cities, for instance, is lower for racialized people and low-income households [36, 37]. Even when social infrastructure is spatially accessible, many residents may not benefit as its use is heavily influenced by neighbourhood perceptions [19, 38, 39]. For example, perceived lack of safety—whether due to poor upkeep and lighting, or social factors like discrimination—can erode social connectedness and exacerbate health inequities for some groups. This includes girls and racialized youth, who use parks less as they transition out of childhood [25, 40]. 

Given these complexities, understanding how social infrastructure shapes social connectedness requires attention to both the objective environments and lived experiences of young people, particularly those from structurally marginalized communities. Yet conventional methods in built environment studies (e.g. direct observation, audits) tend to focus on physical features of neighbourhoods, and overlook contextual factors known to influence access and use of social infrastructure [30, 41]. Participatory and mixed methods that integrate data on neighbourhood perceptions are a promising approach for addressing this methodological gap and enabling a more comprehensive assessment of youth-inclusive environments [16, 30]. 

### Aim and theoretical foundations

This paper advances knowledge on how neighbourhood environments shape social connectedness for young people and why. Our findings stem from Youth.hood, a community-engaged research study designed to uncover and address how built environments help or hinder social connectedness for youth in under-served and racialized communities. Integrating methods from participatory action research and citizen science, Youth.hood engaged youth (aged 15–19 years) from Vancouver, Canada, to: (1) Assess features of their neighbourhood environments that impact social connectedness using a smartphone app; (2) Analyze results of their assessments, prioritize neighbourhood issues, and design solutions for addressing them; and (3) Advocate for neighbourhood improvements with researchers, community partners, and city planners.

Participatory citizen science approaches that leverage technology have been effective for capturing diverse perspectives on urban environments and engaging marginalized residents in neighbourhood improvements, including youth [42, 43]. The term “citizen” in this context refers to any resident, regardless of legal status. Participatory citizen science is distinguished by its emphasis on empowering residents to not only systematically gather data but also translate their findings into solutions through applying principles of participatory action research, ultimately engaging participants as co-researchers and agents of change [44]. Our work draws on Youth Participatory Action Research (YPAR) specifically, which emphasizes youth experiences, research *with* (as opposed to *by*) adults, and transformative action for youth and their communities [30]. 

Our work also draws on socio-environmental affordance theory, which conceptualizes neighbourhood built environments according to the developmental needs, experiences, and opportunities they provide [33, 45]. This approach departs from a narrow view of neighbourhoods as strictly physical and functional spaces to consider other contextual factors that shape how youth perceive and interact with their environment [30, 46]. Evaluating and designing built environments through the lens of developmental affordances—such as social interaction, belonging, and other aspects of social connectedness—is relevant to the dynamic ways youth experience and identify preferred spaces [47] and can provide important insights into pathways between place and adolescent health [34]. 

## Methods

### Design

Youth.hood draws on the *Our Voice Citizen Science Research Initiative* developed by the Health Equity Action Research and Technology Solutions (HEARTS) Lab at Stanford University [42]. In keeping with this approach and YPAR principles, youth were engaged as community scientists in the full research process, from data collection to knowledge mobilization. Aided by a neighbourhood assessment app known as the Healthy Neighbourhood Discovery Tool (aka Discovery Tool) [48], this approach combines smartphone-based photovoice with geospatial data collection, quantitative surveys, and participatory analysis and design workshops. The *Our Voice* method has been applied in projects globally across a range of community issues and groups, including youth [49]. The study was approved by the Research Ethics Board at Simon Fraser University and the Vancouver School Board Research Committee.

### Setting

Youth.hood focuses on three adjacent neighbourhoods in Vancouver, Canada—Sunset, Victoria Fraserview, and Killarney—collectively known as South Vancouver. The project grew out of an existing community-engaged research collaboration between our research team and the South Vancouver Neighbourhood House (SVNH), an organization that delivers programs and services to South Vancouver residents. Our earlier research with SVNH revealed inequities and unmet needs with respect to the funding and allocation of social infrastructure in South Vancouver, including services such as health care, and built environment features like transit, bike and walking infrastructure, and parks [50]. Youth.hood was developed to better understand how youth experience these inequities and engage them in solutions.

According to census-based indicators published by the City of Vancouver, South Vancouver neighbourhoods account for one sixth of Vancouver’s population and 22% of its youth; 80% of residents are racialized and 56% are immigrants; and levels of household income and education fall below the city average [51]. Self-reported health and social connectedness in South Vancouver are also among the lowest in the city; for example, only 37% of residents in Victoria-Fraserview report having four or more people in their support network, compared to 50% in the city overall [52]. 

### Participants

A total of 42 young people were recruited as community scientists. Eligibility criteria included the following: (1) aged 15–19 years; (2) living or attending school in South Vancouver; (3) able to walk or roll (e.g., bike, wheelchair, skateboard) through the community for up to an hour; (4) able to read and speak English. Youth were recruited through secondary schools, community centres, and SVNH programs via social media, email, posters, and in-person promotion. At the start and end of the study, the youth completed an online survey that collected data on: demographics; self-rated health; [53] social connectedness; [54, 55] self-efficacy and neighbourhood perceptions (see Supplementary Material 1) [56]. Youth received a living wage ($25/hour CAD), in cash or gift cards, for their time spent on study activities.

### Procedures

Youth.hood involved three phases, described below, which coincided with each study aim: (1) neighbourhood assessments; (2) participatory analysis and solutions workshop; and (3) knowledge mobilization via a community forum. Activities occurred from September 2021 to September 2022.

#### Neighbourhood assessments

Youth community scientists used the Discovery Tool to walk in their neighbourhood and assess features of their environments that help or hinder social connectedness. The tool enabled users to capture and describe geotagged photographs of neighbourhood features, and visually rate them as good (i.e. an asset) or bad (i.e. a barrier) [57]. Three assessments were done, one in each of the main neighbourhoods.

In advance, youth were instructed to download the app on their device. On the day, youth met at a community location where research staff were on site to welcome them and provide an orientation to the data collection activity. Youth were instructed to take a 30–60 min walk along a route of their choosing and use the Discovery Tool to capture at least 5 pictures in response to the prompt: *“What features of your neighbourhood help you connect with people in your community? What features make social connectedness difficult?”.* Research staff were available to accompany youth for safety and technical support, but the majority of participants chose to walk in pairs without an adult. After their walk, the youth returned to the start location to review and upload their data.

#### Participatory analysis and solutions workshop

Coinciding with our second aim, we hosted a participatory analysis workshop—framed as the Youth.hood Design Jam—where youth were reunited to review their collective data on social connectedness assets and barriers, identify and prioritize themes, and design solutions for neighbourhood improvement. The workshop process was designed using methods from participatory thematic analysis and design thinking (see Analysis). To prepare the youth to engage in analysis and solutions design, the workshop kicked off with a “Cities 101” session led by a youth civic engagement organization, where participants learned about municipal governance and the role of urban planning in shaping cities.

#### Knowledge mobilization

In the final phase, youth had the opportunity to participate as Youth.hood Ambassadors in a community forum co-hosted by Simon Fraser University and the South Vancouver Neighbourhood House. The forum was designed to share research and community perspectives on social infrastructure inequities in South Vancouver, and to engage residents and city builders in dialogue around priorities and solutions. Leading up to the forum, four youth stepped forward as Ambassadors and worked with the research team to further develop themes from their data (see Analysis) and craft a presentation on their findings and calls to action. At the event, the Ambassadors presented to an audience of over 100 residents, elected officials, advocates, and city planners.

### Analysis

Our analytical approach engaged youth in analyzing patterns in their data and translating their findings into solutions in a way that was playful, rigorous, and authentic to their voices. To achieve this, our methods drew from thematic analysis [58–60], photovoice [61], and design thinking [62]. Our process (see Table A1, Supplementary Material 2) follows the six phases of thematic analysis developed by Braun & Clarke [59]. The first three phases took place during the Youth.hood Design Jam and used an inductive, experiential approach. In step one (***Data familiarization***), youth were guided through a process for reviewing, reflecting on, and discussing the photovoice data specific to their neighbourhood. In step two (***Coding***), word clouds were used to facilitate an inductive coding process with youth, helping them to systematically organize, identify, and describe segments of their data. Youth were led through a thematic mapping exercise for step three (***Generating initial themes***) to start identifying and prioritizing shared meanings in their photovoice data. These initial themes informed subsequent phases of analysis and served as problem statements for solutions design.

The final three phases of analysis were undertaken in collaboration with the four Youth.hood Ambassadors. In step four (***Theme development and review***), the research team and Ambassadors returned to the full dataset to assess the fit of initial themes and consider whether they captured what matters most in the data relative to the research question. Four final themes were taken forward. Each Ambassador championed a theme, delving deeper into the data to identify key messages and illustrative photos and quotes, which they presented at the community forum. The analysis was fine-tuned in step five (***Refining***,*** defining***,*** naming themes***) and six (***Writing up and reporting themes***). All photo, text, and survey data were imported into NVivo 12 and recoded by the research team around neighbourhood features and themes identified by the youth. This second cycle of researcher-driven coding took a more deductive and latent approach, interrogating deeper meanings within themes and their relationship to broader literature on neighbourhood environments and social connectedness. In analysis, all names were removed and participants were assigned pseudonyms. Quantitative survey data were analyzed in R (version 1.4).

## Results

### Participant characteristics

Forty-two youth (average age 16.5 ± 0.9 years) participated in at least one component of the study (see Table 1). Participants identified as boys (52%), girls (45%), or transgender (2%). Their racial identities reflect the diversity of South Vancouver overall, with the majority identifying as Chinese (48%) or South Asian (17%). On questions of social connectedness from our pre-survey, 62% of youth rated their sense of community belonging as somewhat/very strong, and 57% had four or more people to confide in.

**Table 1:** Characteristics of youth participants

Characteristics	Total (*n* = 42)	%
**Gender**		
Boy	22	52
Girl	19	45
Trans girl^a^	1	2
**Race** ^**b**^		
Chinese	20	48
South Asian	7	17
Filipino	3	7
Southeast Asian	3	7
White	2	5
Japanese	2	5
Multiple racial groups listed above	3	7
Other racial group not listed above	2	5
**Born in Canada = Yes**	35	83
**Self-rated health**		
Very good to excellent	21	50
Good	15	36
Fair to poor	6	14
**Community belonging**		
Somewhat strong to very strong	26	62
Somewhat weak to very weak	11	26
I don’t know	5	12
**People to confide in**		
None	0	0
1 to 3	12	29
4 to 6	18	43
More than 6	6	14
Prefer not to answer	6	14

^a^ Results for this participant have been grouped with girls to maintain privacy

^b^ Racial groups are consistent with census categories used by Statistics Canada [63]

**Fig. 1:**
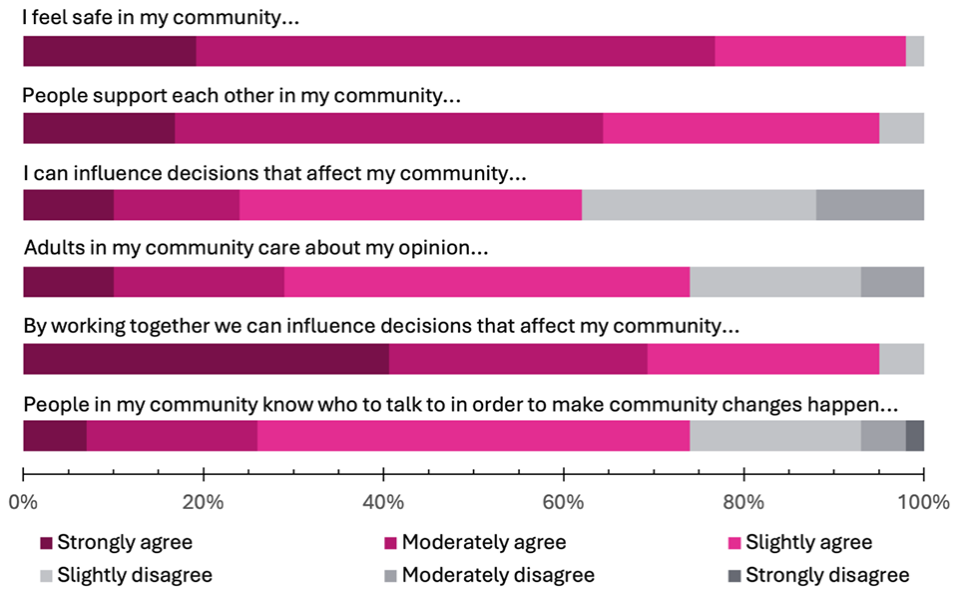
Participant perceptions of social cohesion and self-efficacy

At the start of the project, most youth moderately or strongly agreed with their communities feeling safe (76%) and mutually supportive (64%) (see Fig. 1). While 69% reported moderate or strong agreement with their community having influence over planning decisions, a much smaller percentage agreed with having personal influence over decisions affecting their communities (24%).

### Environmental assets and barriers to youth social connectedness

The youth community scientists captured 193 photos, from which 227 individual features were identified (see Table 2). Youth rated 70% (*n* = 159) as positive (i.e. assets) for social connectedness, 17% (*n* = 39) as negative (i.e. barriers), and 13% (*n* = 29) as having both positive and negative aspects (e.g. parks perceived as poor quality due to litter or lack of upkeep).

**Table 2:** Frequency [n(%)] of neighbourhood features as captured by youth community scientists

Feature	Asset	Barrier^g^	Both	Total
Parks and nature	39 (17)	3 (1)	8 (4)	50 (22)
Neighbourhood aesthetics^a^	18 (8)	14 (6)	5 (2)	37 (16)
Streets and sidewalks^b^	10 (4)	14 (6)	5 (2)	29 (13)
Food outlets^c^	25 (11)	0 (0)	2 (1)	27 (12)
Schools	12 (5)	1 (0)	1 (0)	14 (6)
Public transit	5 (2)	2 (1)	3 (1)	10 (4)
Shops and shopping districts	8 (4)	0 (0)	1 (0)	9 (4)
Public libraries	8 (4)	0 (0)	0 (0)	8 (4)
Community centres	6 (3)	2 (1)	0 (0)	8 (4)
Benches	6 (3)	0 (0)	1 (0)	7 (3)
Boulevards^d^	6 (3)	0 (0)	1 (0)	7 (3)
Clubs	4 (2)	0 (0)	0 (0)	4 (2)
Churches	3 (1)	0 (0)	0 (0)	3 (1)
Unknown^e^	2 (1)	1 (0)	2 (1)	5 (2)
Other^f^	7 (3)	2 (1)	0 (0)	9 (4)
**TOTAL n (% of total features)**	**159 (70)**	**39 (17)**	**29 (13)**	**227 (100)**

^a^ includes cleanliness, upkeep, streetscapes, scenery, public art, placemaking

^b^ includes infrastructure for active transport and traffic noise

^c^ includes restaurants, cafes, convenience stores, food retailers or grocers

^d^ verges between the street and sidewalk

^e^ feature could not be deduced from photo or text description

^f^ grouping of all features where *n* < 3

^g^ shortage or inadequacy in the feature listed

The most frequently reported assets were parks and nature (*n* = 39, 17%) and food outlets (*n* = 25, 11%). Assets were mostly consistent across genders, with the exception of benches and boulevards, which girls captured more. Neighbourhood aesthetics were identified as both positive and negative; assets (*n* = 18, 8%) included public art and street greening, while barriers included litter, vandalism, and lack of upkeep (*n* = 14, 6%).

Other common barriers were inadequate streets and sidewalks (*n* = 14, 6%), specifically lack of infrastructure for walking and biking, traffic-related noise and safety risks, and poor road and sidewalk conditions.

### Thematic summary

Youth identified and prioritized four themes from their collective neighbourhood assessments. These themes (summarized in Table 3 and detailed throughout this section) illustrate what features matter most to their connectedness, and why. Examples of photos corresponding to quotes for each theme are provided in Table A2 (see Additional file 2).

**Table 3:** Summary of themes

Theme		Description
1	Connecting through mobility: The fun and functionality of getting around without a car	Active travel and transit are both modes of transport and innately social activities. Getting around without a car easily, safely, and enjoyably is hindered by inadequate infrastructure and traffic noise.
2	The power of aesthetics: Mediating connections to people and place	Neighbourhood aesthetics impact both physical and emotional connectedness. Attractive neighbourhood features facilitate connection, while poor upkeep is a barrier, making spaces feel unsafe and uninviting.
3	Retreating to connect: Seeking out social and restorative spaces for all	Youth enjoy the bustle of city life but also need spaces to retreat with close friends and family. They value spaces, both formal and informal, that integrate nature and cater to a variety of interests and age groups, not just adolescents. Lack of youth-inclusive amenities, lighting, and weather protection in public spaces are barriers to connection.
4	Under-resourced, not under-valued: Uncovering assets for sociocultural connection	Despite living in under-resourced neighbourhoods, youth captured an array of assets in their communities that matter to their individual, community, and also cultural connectedness.

#### Connecting through mobility: the fun and functionality of getting around without a car

A central theme emphasized by youth was that their social connectedness is closely intertwined with their mobility, specifically their ability to easily, safely, and enjoyably get around without a car. Collectively, youth conveyed how public transit and active travel—including walking, biking, and skateboarding—are methods for reaching the people and places that matter to their connectedness, and innately social activities. Multiple narratives point to this notion of active transport and transit as both functional *and* fun. For instance, in describing their photos of bus stops, Lily explained *“…transit takes me places I want to go…[it] makes my life better because it brings me closer to friends and people I care about”*, while Aisha shared how *“(The bus stop) is good to bring people together…if you have friends with you it’s so much fun because you talk while you’re waiting.”*

Youth described how walking and biking facilitate not only physical connection, but also their emotional connection and wellbeing. Biking was positively characterized as helping to *“connect communities that are a little further from each other”*, *“to relax”*, and have a *“sense of security”*. After capturing a photo of a bustling shopping street in South Vancouver, Meera commented:

I feel strangely safe when I walk on Fraser Street. Seeing so many people walking by and enjoying their lives is wonderful.

While the above perspectives speak to the social benefits of active transportation and transit for youth, participants captured more barriers to their mobility than assets. Youth emphasized three transportation-related features that hinder their social connectedness the most: inadequate quantity of active transportation and transit infrastructure, poor quality of infrastructure, and traffic noise. For example, in describing their photo of a popular walking route between a high school and a nearby park that has no sidewalk, Priya explained: *“People have to walk on the road*,* which becomes dangerous as cars come by. It becomes incredibly difficult to walk and talk at the same time.”* Other youth shared similar experiences about the lack of bus routes to their homes and the shortage of space on the road to bike safely.

Regarding infrastructure quality, multiple youth captured how poorly maintained roads and crumbling sidewalks hinder the ability to comfortably and safely get around, whether for transport or leisure. Priya pointed out how *“Old people especially are affected by [bad sidewalks] as this limits them from going out and socializing with their friends and family”.* Similarly, Morgan shared a story about skateboarding to get bubble tea with a friend that ended in them (and their tea) being *“taken out”* by a large pothole:

I recently went [skateboarding] here with my friend and it was super, super fun….but….the road was in bad condition. He tried to do a slide and his hand got caught and he fell, basically. I enjoy [skateboarding on] these hills…but I would like them to be [re]paved. It promotes the use of vehicles that aren’t cars. Because nice pavement means…you can ride on it, walk nicely, it looks nice in general. And it’s safe….

Traffic-related noise was also a barrier to social connection. Attempting to walk and talk against a backdrop of noise from heavy traffic cutting through their communities was described by youth as *“annoying”*,* “uninviting”*, and the *“one thing”* they don’t like about their neighbourhood.

#### The power of aesthetics: mediating connections to people and place

Neighbourhood aesthetics also played a powerful role in shaping individual and community connectedness for youth, both positively and negatively. While some youth captured *“good places”* for social interaction, for many participants the positive impact of aesthetics on social connectedness was distinctly more emotional than physical. “*Every time I look at them I can’t help but stare in awe*,*”* shared Meera in response to their photo of street trees. Public art, both commissioned and resident-led (for example, a DIY bird feeder on a street boulevard), evoked a similar emotional response from participants, who described these features as *“eye candy”*, *“heart-warming”*, and a way to *“strengthen our sense of community”*. Implicit in narratives on the positive impact of aesthetics was an emphasis on emotional connections to both people and place. A sense of community pride and identity was evident in several of the youths’ explanations. For instance, two youth captured pictures of the sunset viewed from the neighbourhood main street *“because it’s what [our community] is known for”.* In another photo of a street named to commemorate Vancouver’s oldest Sikh society, Chloe shared:

This street always reminds me that I live in a culturally diverse part of Vancouver. Before Covid there would be this huge Vaisakhi celebration every year around my mom’s birthday. Even though we didn’t celebrate it, we’d go out as a family and meet with friends to join the community celebrations!

Overshadowing these assets were reports of neighbourhood neglect, which youth perceived as making their communities appear unsafe and uninviting. Close to half of the aesthetic features captured by youth were rated as barriers, the most common being garbage, disrepair, and vandalism. They took photos of garbage in streets, parks, and around bus stops, describing litter as *“such a big problem”* that deters them and others from using spaces. Poor maintenance like worn street signs, unkept parks, and rusted playground equipment also deterred youth from wanting to spend time in places. For instance, Leo explained how minor upkeep can make a major difference to youth’s desire to use spaces:

Honestly it does sound pretty trivial but the fact that this basketball court has nets on the hoops means a lot. The experience and the sound of having a net on the basketball hoop makes playing at this specific court a lot more enjoyable compared to one that doesn’t.

Youth’s descriptions of vandalism illustrate how lack of upkeep misrepresents the positive identity they associate with their neighbourhood, leaving a wrong impression of it being unsafe and uninviting. Multiple youth captured one particular house that had been abandoned and graffitied, explaining how it *“doesn’t give a good vibe”*,* “isn’t very inviting”* to walk around at night, and “*makes [the neighbourhood] seem scary when in reality it’s not.”*

#### Retreating to connect: seeking out social and restorative spaces for all

A third theme underscored by youth was that, while they enjoy the bustle of city life, they also desire spaces to retreat to with family and friends that are quieter and flexible to a variety of interests and age groups, not only adolescents. Parks and green spaces surfaced as essential for connection by providing opportunities to play sports with others, *“hang out”*, hold family or community gatherings, or *“just talk”*. Youth perceived parks and natural spaces as both sociable and restorative. *“Having both [bustling spots and quiet*,* relaxing spots] is important to create a community between friends and others”*, remarked Taylor. Reflecting on a local park they cherish, Taylor commented:

[My friends and I] have spent many afternoons here as well as holidays. The greenery and open space make it a calming place to hang out. I think even more green spaces in our neighbourhood would be a good way to strengthen our community spirit.

Assets captured by youth included designated parks with a mix of amenities—like sports facilities, seating, and open spaces—as well as smaller, more informal public spaces. Multiple youth voiced their attraction to and appreciation for street boulevards, some equipped with benches, describing them as restful and convenient spaces to *“connect and interact with people in our community”*,* “relax from the busy street”*,* “enjoy good food and conservation with friends*,* families*,* and neighbours”*, and *“enjoy a decent view down the street”.*

Several youth expressed a sense of nostalgia for parks and public spaces they frequented in their childhood. For example, youth recounted memories of going to their local park “*after dinner with a group of [elementary school] friends to play [games]”*, going to block parties when they were younger *“where all the neighbours would bring and share food”*, and benches where they *“first hung out with a cool friend”.* Relatedly, many participants described playgrounds as *“good for little kids”* rather than assets for youth more generally. On the surface, these patterns demonstrate the tendency for youth to consider the needs of other groups in the community (in this case, younger children), but more implicitly, they signal a lack of amenities relevant to the lives and needs of adolescents. In some cases, the absence of appealing amenities was captured by youth explicitly, despite the neighbourhood assessment process guiding them towards assessing what’s existing in their neighbourhood—not necessarily what’s missing. For example, after snapping a picture in a particularly under-resourced part their neighbourhood, Jason reflected:

This community is a little bit old. There is a little bit of vandalism on the poles and stuff like that…it isn’t very inviting. I don’t particularly like this location…it feels undeveloped and a little bit unsafe. For a community of this size…you would think there is better assistance. Maybe even a neighbourhood house or community centre. It’s not really a space for youth or somewhere I would want to hang out.

#### Under-resourced, not under-valued: uncovering assets for sociocultural connection

Although their neighbourhoods are objectively under-resourced, 70% of features captured by youth were considered assets (see Table 2). When describing why these assets matter—beyond helping them gather, meet new friends, engage in the community, and feel a sense of belonging—youth emphasized the importance of diversity and sociocultural connection. Multiple youth captured photos of multicultural eateries and grocers, describing how *they “deepen my relationship to where I was born”*, *“allow me to explore other cultures”*, and *“connect me to my culture even if I’m not (in the) Philippines”*. Leo’s reflections about a popular bubble tea shop illustrate how local food outlets meant more to youth than simply places to eat:

This location is quite popular because it’s close by different high schools and places where youth frequently visit. The fact that it’s a bubble tea shop and (South) Vancouver is predominantly made up of an Asian demographic…I think it draws a lot of popularity from youth who want to gather and they see that their own culture is being represented.

Youth’s emphasis on diversity and sociocultural connection extended to features beyond food assets. Reflecting on their photo of a lively multicultural shopping street, Aisha commented *“(This) street alone feels like a community in which you belong because of the diverse stores they have opened*,* and because of the diversity of the people walking around the streets”.* They shared a similar sentiment about the local public library, a featured mentioned by many as an important anchor space for social interaction and inclusion, for themselves and the community at large:

The library is an excellent place that helps you feel like you fit into your community. You get to interact with others, find other readers who love to read the same genre as you, and best of all there are tables and chairs which you can use to study or complete a project with friends. Also the library is accessible for all, any age…this helps create connections with a diverse group of people.

## Discussion

Harnessing citizen science and a participatory action research approach, this paper explores how neighbourhood environments shape social connectedness for young people and why, based on the lived experiences of youth from under-resourced and racialized communities. Through neighbourhood assessments and participatory analysis, youth identified four themes: (1) Connecting through mobility: The fun and functionality of getting around without a car; (2) The power of aesthetics: Mediating connections to people and place; (3) Retreating to connect: Seeking out social and restorative spaces for all; and (4) Under-resourced, not under-valued: Uncovering assets for sociocultural connection.

Despite living in neighbourhoods with inadequate social infrastructure, youth identified a range of features that facilitate individual and community connection. Many were formal public and commercial spaces, including shops and shopping districts, libraries and community centres, food outlets, and parks with recreational amenities [13, 24, 64–68]. Our study extends research documenting the importance of these spaces to facilitating social interaction, support, and a sense of community [69, 70] by adding to the limited evidence on how racialized youth experience and value formal social infrastructure [9, 70, 71]. 

Our findings also highlight the role of more ambiguous elements of neighbourhoods in facilitating connectedness for youth, in particular informal or liminal green spaces that enable unconstrained leisure, socialization, and nature contact [72–74]. Several youth voiced how street boulevards, whether equipped with basic benches or simply grass, serve as spaces for impromptu relaxation and interactions with friends and neighbours. While the health benefits of formal green spaces are well-documented [17], we contribute to emerging research on the use and perceived value of non-park green spaces in under-resourced communities, and how these spaces may help level spatial inequities in green space accessibility [75–78]. Additionally, activating informal green spaces—for example, through bylaws that allow for spontaneous placemaking and seating designed for interaction [64, 79]—could be a leverage point for mitigating social inequities, in particular by improving green space accessibility for girls, racialized people, and others who may feel excluded or unsafe in formal public spaces [40, 80]. 

The extent of assets uncovered in our study illustrate how youth in underserved communities are making the most of the spaces available to them. Yet, as detailed in our third theme, the perceived lack of relevant and appealing spaces for youth still surfaced as a barrier to their connection. Many positive narratives of parks and other public spaces spoke to their value in childhood, not adolescence. This experience—where the needs and interests of youth are often an afterthought, stuck in between planning processes that revolve around children and adults—is consistently raised in research and dialogue on youth-friendly communities. We echo previous calls for local environments that go beyond meeting youth’s basic needs to inspire and engage them as welcomed users [66]. 

Beyond identifying discrete neighbourhood assets important to youth’s social wellbeing, our findings shed new light on the nuanced ways that mobility and neighbourhood aesthetics influence individual and community connection. Our youth’s experiences were consistent with established evidence showing how inadequate active transportation and transit infrastructure and poor neighbourhood upkeep limit the ability to access, use, and linger in social infrastructure [39, 81, 82]. Studies in lower socioeconomic areas have highlighted how aesthetic barriers identified in our study—poor lighting, vandalism, disrepair, litter, and traffic—intersect psychosocial and structural factors to influence negative perceptions of neighbourhood identity, attractiveness, safety, and trust [11, 16, 81, 83, 84]. 

Enabling independent mobility is a defining element of child and youth-friendly environments [29, 66]. Our study adds to past work by illuminating the sociability of active travel and transit use [85–88]. Youth’s assertions about the “fun and functionality” of getting around without a car echoes arguments that travel has both a utilitarian and intrinsically social or relational dimension, through the potential for spontaneous and diverse social interactions, and sense of connection to people, places and community [29, 85]. From a research and planning standpoint, our findings bolster the case for attending to transportation infrastructure—whether bus stops, bike lanes, streets, or sidewalks—as more than strictly functional spaces, but as a type of social infrastructure in their own right [9, 13, 79, 89]. 

In voicing why particular features helped or hindered their connectedness, youth identified a range of social affordances that reinforce, but also go beyond, the importance of “hanging out”. Framed through the lens of developmental affordances, youth described their local environments as affording (or denying) opportunities for interaction, informal and organized play, meeting new people, and retreating and relaxing with close friends and family. These findings align with work on the role of the built environment in fostering both socialisation and restoration [24, 33, 90, 91]. Youth also identified affordances beyond individual-level connection, notably, a sense of community belonging, attachment, and cultural connection. Youth’s positive narratives about the diversity, cultural identity, and belonging they experience in everyday functional spaces (e.g., streets, libraries, bubble tea shops) signifies how the shared use of social infrastructure can generate a common sense of tolerance, community pride, and “togetherness” [12, 69]. Other work has similarly identified cultural diversity as a source of comfort and belonging for young people [9]. While racial tension was not referenced in our study, there is substantial evidence of exclusion and discrimination experienced by youth in ethnically diverse neighbourhoods [27, 90, 92, 93]. 

### Implications and impacts

Locally, our community forum sparked dialogue and urgency among city staff and officials to address the neighbourhood inequities disadvantaging South Vancouver. After a year-long advocacy effort, Vancouver City Council approved a motion in June 2023 to improve social infrastructure in the area [94]. Its actions include a direction that staff use Youth.hood’s results to inform engagement, neighbourhood improvement, and policy and funding decisions. Our project offers city planners rich locational data on social connectedness assets, barriers, and solutions directly through the eyes of youth. This unique integration of micro-scale spatial data with experiential knowledge is valuable for guiding place-based improvements for under-served communities [43]. 

Youth.hood’s findings illuminate broader guiding principles for creating connected and healthy communities with and for youth. Despite youth living in neighbourhoods suffering from disinvestment, a positive viewpoint is still evident in their narratives. This reinforces recommendations for asset-based planning approaches that support communities to revitalize from the inside out by building upon their existing resources and ways of thriving [79, 95]. Secondly, our study justifies calls to shift from top-down consultation towards more citizen-initiated models of co-production [35, 64, 96]. Finally, countering the practice of designing age-segregated spaces, youth voiced their desire for intergenerational spaces that are inclusive to a diversity of interests and age groups [25, 64, 97]. 

Further, our research process provided a gateway for youth to influence a system in which they are traditionally disempowered. Youth had the opportunity to show agency in response to the environmental inequities they face—collecting and analyzing their own data, building their civic literacy, setting priorities and solutions, and voicing their needs directly to decision-makers, including at City Hall. Our process and impacts reinforce YPAR and citizen science as transformative both for communities and the participants [42, 43, 98, 99]. 

### Limitations and future research

Youth’s neighbourhood assessments were based on one-hour walks with fixed start and end locations, and as such, they do not capture the entirety of relevant assets and barriers in their communities. The locations were selected for coverage across South Vancouver while prioritizing areas that, according to our community partner, are the most under-resourced. Future research may add a longitudinal component that invites participants to assess features over time (e.g., a typical week, or across seasons). In addition, our findings may not be generalizable to youth in all under-resourced neighbourhoods, or all youth in South Vancouver. The youth’s demographics reflect the diversity of their neighbourhoods, however, certain groups may be underrepresented, for example, newcomer youth who did not meet the eligibility requirement to read and speak English.

## Conclusion

This paper sheds light on the vital ways that neighbourhood built environments shape social connectedness for young people. Specifically, we leveraged methods from citizen science and participatory action research to amplify the experiences of youth from racialized communities disadvantaged by inadequate social infrastructure. Our findings add to mounting literature underscoring the importance of active transportation and transit infrastructure, appealing neighbourhood aesthetics, and inclusive and flexible green spaces—both formal and informal—as key features of socially connected cities for youth. Their experiences point to the potential of neighbourhood planning in facilitating multiple affordances for healthy social development, spanning individual, community, and cultural connectedness. Additionally, this work demonstrates the resilience and agency of youth in under-resourced settings, and the value of honouring assets, co-production, and intergenerational planning when building youth-friendly communities.

The importance of this research is heightened by the prevalence of isolation and loneliness among young people, and the risk this poses to individual and community health. Engaging young people as both community experts and advocates is essential to capitalizing on the potential of place-based interventions for advancing healthy and youthful cities. As illustrated in this work, participatory citizen science can help to achieve this, leading to transformative change both for participants and their communities.

## Electronic supplementary material

Below is the link to the electronic supplementary material.

Supplementary Material 1: Survey questions

Supplementary Material 2: Table A1 (detailing the participatory analysis process) and Table A2 (outlining illustrative photos and quotes by theme)

## Data Availability

Data generated during the current study are not publicly available due to privacy reasons. De-identified photovoice and survey data may be available from the corresponding author on reasonable request.

## Abbreviations

YPAR: Youth participatory action research
SVNH: South Vancouver Neighbourhood House
