# Supplementary material for: (Dis)connected by design? Using participatory citizen science to uncover environmental determinants of social connectedness for youth in under-resourced neighbourhoods
Source: BMC Public Health. 2024 Nov 11;24:3104. doi: 10.1186/s12889-024-20597-4 (PMC11552136; doi:10.1186/s12889-024-20597-4)
Supplement: Supplementary file 1 — Supplementary Material 1: Survey questions [file 12889_2024_20597_MOESM1_ESM.pdf]

Additional file 1 – Survey questions

DEMOGRAPHICS:

1. **What is your current gender identity?**
    - Woman
    - Man
    - Trans woman
    - Trans man
    - Genderqueer/Gender non-conforming
    - Prefer not to answer
    - Different identity (please specify)

2. **To which ethnic or cultural groups do you belong?**
    - Aboriginal
    - White
    - South Asian (e.g., Indian, Pakistani, Sri Lankan, etc.)
    - Chinese
    - Black
    - Filipino
    - Latin American
    - Arab
    - Southeast Asian (e.g., Vietnamese, Cambodian, Laotian, Thai, etc.)
    - West Asian (e.g., Iranian, Afghan, etc.)
    - Korean
    - Japanese
    - Other (please specify):
    - I don't know/prefer not to answer

3. **Were you born in Canada?**
    - Yes
    - No

HEALTH:

4. **Compared to others of your own age, how would you rate your health?**
    - Excellent
    - Very Good
    - Good
    - Fair
    - Poor

SOCIAL CONNECTEDNESS:

5. How would you describe your sense of belonging to your local community?

- Very strong
- Somewhat strong
- Somewhat weak
- Very weak
- I don't know

6. How many people do you have in your network that you could confide in, tell your problems to, or call when you really need help?

- 0
- 1 to 3
- 4 to 6
- More than 6
- Prefer not to answer

SOCIAL COHESION and SELF-EFFICACY

7. Please rate your level of agreement with these statements about your community:

a. I feel safe in my community.
b. People support each other in my community.
c. I can influence decisions that affect my community.
d. Adults in my community care about my opinion.
e. By working together with others in my community, we can influence decisions that affect my community.
f. People in my community know who to talk to in order to make community changes happen.

[Response options for each question…]

- Strongly disagree
- Moderately disagree
- Slightly disagree
- Slightly agree
- Moderately agree
- Strongly agree
