# Supplementary material for: (Dis)connected by design? Using participatory citizen science to uncover environmental determinants of social connectedness for youth in under-resourced neighbourhoods
Source: BMC Public Health. 2024 Nov 11;24:3104. doi: 10.1186/s12889-024-20597-4 (PMC11552136; doi:10.1186/s12889-024-20597-4)
Supplement: Supplementary file 2 — Supplementary Material 2: Table A1 (detailing the participatory analysis process) and Table A2 (outlining illustrative photos and quotes by theme) [file 12889_2024_20597_MOESM2_ESM.pdf]

Additional file 2

Table A1. Participatory analysis process [adapted from Braun and Clarke(2001)]			
Phase and objective		Tools	Activities
1	**Data familiarization:** Immerse youth community scientists in their collective neighbourhood assessment data	**Photocards**: Neighbourhood assessment data were processed into individual reports, auto generated by the *Our Voice* platform, which present each photo with its description, geo-location, and rating. Reports were printed onto individual cards, numbered, and grouped by neighbourhood.<br><br>**Youth.hood map:** Locations of all neighbourhood features were plotted onto a map of South Vancouver and printed in large format. Each pin was colour-coded by rating (assets, barriers, both) and numbered to correspond to its photocard.	At the Youth.hood Design Jam, youth were divided into neighbourhood teams and supported by a facilitator and notetaker. Youth were invited to individually scan the photocards and pick two photos (one asset, one barrier) that resonated most with their lives and experience in the neighbourhood. Youth then shared one of their photos with the group and critically reflected on its importance and meaning, using discussion prompts based on the SHOWeD framework. Next, youth shared any important social connectedness assets or barriers they felt were missing in the data. Additional features were discussed among the youth and added to the Youth.hood map using post-it notes.
2	**Coding:** Guide youth through an open, inductive coding process to systematically organize, identify, and describe segments of their data.	**Word clouds:** Each team of youth received word clouds visualizing the collective descriptions of social connectedness assets and barriers in their neighbourhood.	Youth were asked to review the word clouds independently and circle terms that resonate with them the most. Next, youth worked as a team to review the photo cards for their neighbourhood and tag (i.e. code) them with short descriptions that capture their relevance or meaning, using terms in the word cloud as a guide if desired.
3	**Generation of initial themes:** Steer youth through a thematic mapping exercise to identify and prioritize shared meanings in their photovoice data.	**Tagged photo cards:** Coded by youth in step 2.<br><br>**"How Might We" questions:** A design thinking tool for reframing issues into solvable problems (e.g. "How might we make parks cleaner and safer"?).<br><br>**"Dotmocracy":** An established method for collaborative priority setting and decision	Youth clustered the tagged photocards into thematic categories, then ranked the assets and issues within each theme based on importance and relevance to the research question. Next, youth picked their top three themes and gave each a short title that captures its meaning. To shift into solutions design, each team turned their top themes into How Might We questions, then worked individually to brainstorm solutions for each. Next, using "dotmocracy", youth voted for

		making, where participants vote used to describe vote on their preferences using a limited number of dot stickers.	three solutions they perceived as most feasible and potentially impactful. Finally, youth debriefed the top solutions and discussed steps required for their implementation.
4	**Theme development and review** Assess the fit of initial themes, consider whether they capture what matters most in the data relative to our research question, identify final themes.	**Data briefs:** A summary of initial themes, solutions, and field notes coming out of the Youth.hood Design Jam (Step 1-3).	Youth.hood Ambassadors worked with the research team to develop and review a final set of four themes, based on the initial themes identified at the Design Jam. Ambassadors: (1) selected a theme to champion; (2) reviewed the data brief for their theme and considered whether it captures key patterns; (3) wrote up key messages for their theme to voice to city planners and residents; and (4) selected photos/quotes that best illustrate their theme. Ambassadors invested 5 hours into this process.
5	**Refining, defining, naming themes** Interrogate deeper latent meanings around themes and their relationship to broader literature on the relationship between built environments and youth social connectedness.	NVivo 12<br>R (version 1.4)	To fine-tune the analysis, a second cycle of researcher-driven coding was completed. All photovoice was imported into NVivo and coded by the research team to organize photos and associated descriptions around the neighbourhood features and themes identified by youth. Photos capturing more than one barrier or asset were assigned multiple content codes. Descriptive analysis of pre-survey data shared by the youth community scientists was analyzed using R.
6	**Writing up and reporting themes** Support youth to voice their findings and needs to key actors.	Presentations to residents, community leaders, City of Vancouver staff, Mayor and Council, Provincial Ministers<br>ArcGIS StoryMap<br>Dissemination toolkit	Ambassadors worked with the research team to package their messages and photos into a compelling presentation, which they delivered at the community forum on September 17, 2022.<br>Ambassadors delivered additional presentations to City of Vancouver staff (Apr 2023), Mayor and Council (June 2023), and Provincial Ministers (Oct 2023).<br>An interactive mapping tool that integrates spatial and photovoice data is in development, to help guide local place-based improvements.

Table A2. Illustrative photos and quotes by theme

Theme and description		Example photo	Corresponding quote
1	**Connecting through mobility: The fun and functionality of getting around without a car**<br><br>Active travel and transit are both modes of transport and innately social activities. Getting around without a car easily, safely, and enjoyably is hindered by inadequate infrastructure and traffic noise.	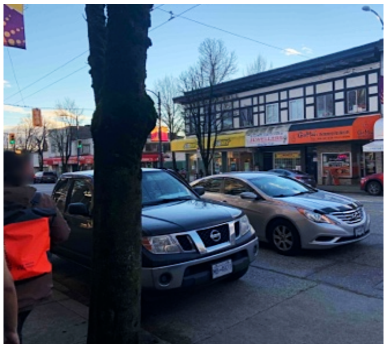	"I feel strangely safe when I walk on Fraser Street. Seeing so many people walking by and enjoying their lives is wonderful."
		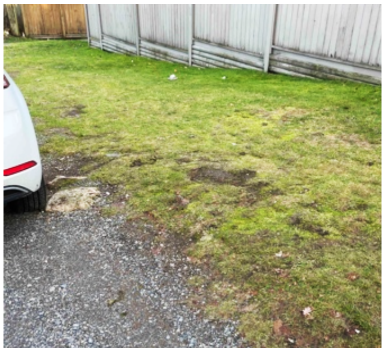	"No sidewalk, so people have to walk on the road, which becomes dangerous as cars come by. It becomes incredibly difficult to walk and talk at the same time."
2	**The power of aesthetics: Mediating connections to people and place**<br><br>Neighbourhood aesthetics impact both physical and emotional connectedness. Attractive neighbourhood features facilitate connection, while poor upkeep is a barrier, making spaces feel unsafe and uninviting.	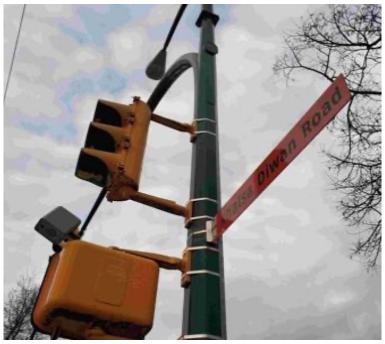	"This street reminds me that I live in a culturally diverse part of Vancouver. Before Covid there would be this Vaisakhi celebration every year. We'd go as a family and meet with friends to join the celebrations!"
		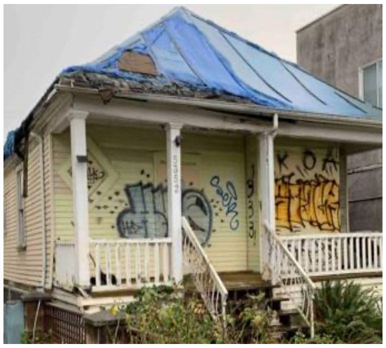	"Buildings like these are what really makes [the neighbourhood] seem scary when I know in reality it's not. It's hard to ignore. If the city could do something about the property [here] or in surrounding areas it would...make youth a lot more comfortable."

Table A2 (continued). Illustrative photos and quotes by theme

3	**Retreating to connect: Seeking out social and restorative spaces for all**<br><br>Youth enjoy the bustle of city life but also need spaces to retreat with close friends and family. They value spaces, both formal and informal, that integrate nature and cater to a variety of interest and age groups, not just adolescents. Lack of youth-inclusive amenities, lighting, and weather protection in public spaces are barriers to connection.	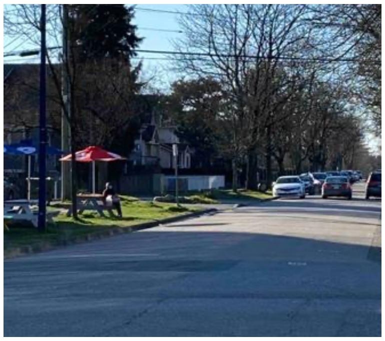	"The benches on Fraser [St] help us connect with our community, enjoy good food...have a conversation with friends, family and neighbours. It's in a place where people walk so [they] have the chance to sit and relax."
		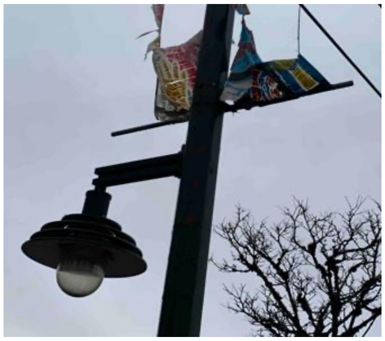	"This community is a little bit old. There is a little bit of vandalism on the poles and stuff like that...it isn't very inviting. I don't particularly like this location...it feels undeveloped and a little bit unsafe. For a community of this size...you would think there is better assistance like programs. Maybe even a neighbourhood house or community centre. It's not really a space for youth or somewhere I would want to hang out."
4	**Under-resourced, not under-valued: Uncovering assets for sociocultural connection**<br><br>Despite living in under-resourced neighbourhoods, youth captured an array of assets in their communities that matter to their individual, community, and also cultural connectedness.	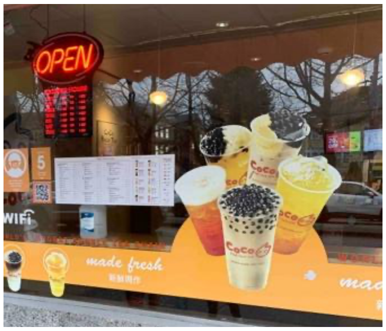	"The fact that it's a bubble tea shop...I think it draws a lot of popularity from youth who want to gather and they see that their own culture is being represented."
		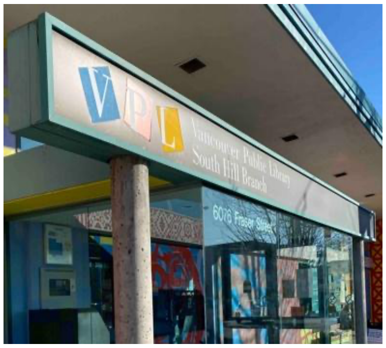	"The library helps you feel like you fit into your community.....The library is accessible for all, any age...this helps create connections with a diverse group of people."
